# Genetic Variants of CD40 Gene Are Associated with Coronary Artery Disease and Blood Lipid Levels

**DOI:** 10.1155/2016/1693619

**Published:** 2016-04-20

**Authors:** Liting Zhou, Lin Xie, Dongchun Zheng, Na Li, Jian Zhu, Shuyue Wang, Bo Li, Lin Ye

**Affiliations:** ^1^Department of Occupational and Environmental Health, School of Public Health, Jilin University, Changchun 130021, China; ^2^Department of Emergency, China-Japan Union Hospital, Jilin University, Changchun 130021, China; ^3^Department of Epidemiology and Statistics, School of Public Health, Jilin University, Changchun 130021, China

## Abstract

*Objectives*. The present study aimed to evaluate the effect of* CD40* and* CXCR4* genes polymorphisms on CAD susceptibility and the blood lipid levels and history of cardiovascular risk factors in a Chinese Han population.* Materials and Methods*. A total of 583 unrelated patients with CAD and 540 controls were recruited. Two tag SNPs (rs4239702 and rs1535045) at the* CD40* locus and one tag SNP (rs2228014) at the* CXCR4* locus were genotyped using the SEQUENOM Mass-ARRAY system.* Results*. After adjusting the risk factors, the frequency of rs1535045-T allele was also higher in patients than controls. Haplotype analysis showed that the rs4239702(C)-rs1535045(T) haplotype was associated with CAD. People with rs4239702-TT genotype had higher blood lipid levels in case group while it was not in the control group. History of cardiovascular risk factors showed no association for the three SNPs in case group and control group.* Conclusions*. rs1535045 in* CD40* gene is likely to be associated with CAD in the Chinese Han population. rs4239702(C)-rs1535045(T) haplotype was associated with CAD. Only in CAD patients, the blood lipid level of patients with rs4239702-TT genotype was higher than other patients.* CXCR4* gene may not relate to CAD.

## 1. Introduction

Coronary artery disease (CAD) is a complex disease and it has become the leading cause of death of human diseases worldwide [1–4]. There is no doubt that a genetic factor, which accounted for 30%~60% of the risk of CAD [5, 6], and the traditional risk factors, such as age, male gender, smoking, drinking, obesity, hypertension, hypercholesterolemia, and diabetes mellitus, contribute to the pathogenesis of CAD.

Vascular function is an important pathophysiological factor in cardiovascular diseases, and is influenced by many factors. Evidence suggested that inflammation may also be a risk factor for CAD [7]. Inflammation in the vessel wall plays an essential role not only in the initiation and progression of the atherosclerotic lesion, but also in the destabilization and acute rupture of plaques that occur during acute myocardial ischemic events [8–10]. Atherosclerotic lesion and acute myocardial ischemia share the same mechanism with CAD. Thus inflammation is considered to be one of the mechanisms of CAD.

As a consequence of the expanding research, numbers of inflammatory marker tests ordered by clinicians for cardiovascular disease (CVD) risk prediction have grown rapidly. Up to now, several new nontraditional markers such as CD40-CD40 ligand system and chemokine receptors seem to be significant features of cardiovascular diseases [11, 12].

It is now generally accepted that CD40-CD40 ligand (CD40L) interaction is a main determinant of the proatherogenic phenotype [13]. Studies suggested that patients with unstable CAD had an elevation of serum-soluble CD40L levels [14–16]. The genetic variants of* CD40* gene may influence the efficiency of* CD40* gene translation and may determine an individual's susceptibility on acute coronary syndrome [17, 18]. CXCR4 is required for neovascularization and is also involved in inflammation. Patients with CAD were observed a reduced expression of* CXCR4* gene [19].

However, the relationship between* CD40* and* CXCR4* gene polymorphisms and CAD is poorly understood until now. In this study, we strived to investigate the potential genetic role of* CD40* and* CXCR4* in the susceptibility of CAD in a Chinese Han population. In order to take into consideration traditional risk factors affecting genetic analysis in CAD, the present study was therefore undertaken to investigate the combined effect of the two genes and some common risk factors on the development of the disease.

## 2. Materials and Methods

### 2.1. Subjects

A total of 583 unrelated patients with CAD from the First Hospital of Jilin University, Changchun, China, were recruited between 2008 and 2012. All patients had been examined by standardized coronary angiography according to the Seldinger's method, and the results were judged by at least two independent cardiologists. According to the coronary angiographic results, patients diagnosed with CAD had at least one or more major coronary arteries with 50% or greater stenosis. Control subjects (*n* = 540) were randomly selected from the same geographical area in the routine check-up as part of annual body examination, including an electrocardiogram (ECG), chest X-ray, and serum analysis. They were classified as healthy subjects based on their physical examination coupled with the absence of personal or family history and other reasons to suspect CAD. Individuals were excluded from having congenital heart disease, cardiomyopathy, and liver and renal disease.

The demographic and clinical characteristics data were collected through a review of the medical records. All the subjects were given an informed consent and were well told of the study protocol. The study was approved by the ethics committee of Jilin University, Changchun, China. Peripheral venous blood was collected in tubes containing disodium-EDTA (ethylenediaminetetraacetic acid) as an anticoagulant and stored at −80°C until genomic DNA extraction.

### 2.2. Genotyping

To perform genetic analysis, we selected two tag SNPs (rs4239702 and rs1535045) at the* CD40* locus and one tag SNP (rs2228014) at the* CXCR4* locus as genetic markers. The selected SNPs were restricted to frequency of minor allele >10% in the HapMap-CHB database (http://www.hapmap.org/).

Genomic DNA used for SNPs genotyping was extracted from peripheral blood lymphocytes using a DNA extraction kit (TianGen, Beijing, China). SNPs were genotyped using SEQUENOM Mass-ARRAY system with amplification primers and extension primers described in Table 1. For quality control, genotyping was performed without knowledge of the case or control status. 30 random samples were tested in duplicate by different persons, and the reproducibility was 100%.

### 2.3. Statistical Analysis

SPSS16.0 for windows was used for analysis. Data were expressed as percentages of total for categorical variables, or median ± QR and mean ± SD for continuous variables. The statistical analysis on the characteristics of the subjects was performed with Student's *t*-test for the continuous variables with normal distribution (BMI), TC, and TG, while skewed distribution variables were compared by the Mann-Whitney *U* test (age, WHR) and Pearson *χ* ^2^ test for the categorical variables.

The statistical differences between case and control in genotype and allele frequencies were assessed by *χ* ^2^-test. The logistic regression models were performed to adjust the risk factors and calculate the odds ratios (ORs) and 95% confidence intervals (CIs). The statistical analysis on the association of the genotypes with blood lipid level was performed with *F* test and multiple comparisons with Dunnett test. Genotype association for history of cardiovascular risk factors, including diabetes mellitus and hypertension, was performed with *χ* ^2^ test.

The Haploview program (version 4.1) was applied to estimate the linkage disequilibrium (LD) measures (*D*′ and *r*2) between paired SNPs. Haplotype analysis was performed with the unphased program (Version 3.0.12).

## 3. Results

### 3.1. Characteristics of Study Subjects

Table 2 lists the demographic and clinical characteristics of the 583 CAD patients and 540 control subjects. Compared with the control group, the CAD group had more smokers and more individuals with hypertension and with diabetes mellitus. CAD patients also had higher waist-to-hip ratio (WHR). There was no significant difference in the mean age, sex, body mass index (BMI), TC, and TG.

### 3.2. Allele and Genotype Analysis

Tables 3 and 4 summarized the allelic and genotypic frequencies of the SNPs in Chinese CAD patients and controls. As shown, the frequency of rs1535045-T allele was higher in patients than controls (OR = 1.21, 95% CI 1.01–1.45, *P* < 0.05); after adjusting the risk factors (smoking, hypertension, diabetes mellitus, and WHR), the OR is 1.27 (95% CI 1.01–1.59). But there was no significant difference in distribution of genotypic and allelic frequencies of rs4239702 and rs2228014 and in distribution of genotypic frequencies of rs1535045 between cases and controls.

### 3.3. Haplotype Analysis

Analysis with Haploview showed that rs4239702 and rs1535045 were located in a similar LD block (*D*′ = 1.0).

We used unphased program to analyze the relationship between the haplotypes of the CD40 gene and CAD. rs4239702(C)-rs1535045(C) haplotype was selected to be the reference for testing the overall association. As shown in Table 5, there was no significant difference between cases and controls of the overall association, but the rs4239702(C)-rs1535045(T) haplotype was associated with CAD (OR = 1.246, 95% CI 1.014–1.531, *P* < 0.05).

### 3.4. Genotype Association for Blood Lipid

Tables 6 and 7 analyzed the genotype association of the 3 SNPs with blood total cholesterol (TC) and blood triglyceride (TG) levels in CAD patients and controls.  Table 6 showed that CAD patients with rs4239702-TT genotype had higher total cholesterol level than patients with rs4239702-CC genotype (*P* < 0.05), while, in control group, there was no difference between genotypes and blood total cholesterol level (*P* > 0.05).  Table 7 showed that, in control group, people with rs4239702-TT genotype had the lowest blood triglyceride level than people with rs4239702-CT genotype or rs4239702-CC genotype (*P* < 0.05), while, in CAD patients, there was no difference between genotypes and blood triglyceride level (*P* > 0.05).

### 3.5. Genotype Association for History of Cardiovascular Risk Factors

Tables 8 and 9 analyzed the genotype association of the 3 SNPs with diabetes mellitus and hypertension in CAD patients and controls. Neither of them showed association for the three SNPs.

## 4. Discussion

Coronary artery disease is a complex disease, which may be due to genetic and environmental factors interaction. Previous studies have suggested that the smoking, drinking, obesity, hypertension, diabetes mellitus, and circulating blood lipid levels were independent risk factors for cardiovascular diseases [20–22]. In this case-control study, we matched cases and controls by age and sex. Several classical risk factors for CAD were detected as well. There were no significant differences in the BMI, TC, and TG between the cases and controls. That may be caused by our small sample size or by patients taking medicine for lipid-lowering therapy. However, compared with controls, the CAD group had more smokers, more individuals with hypertension and with diabetes mellitus, and higher WHR. Our results confirmed that smoking, hypertension, diabetes mellitus, and central obesity are related to CAD.

Inflammation has been found to be involved in the formation, development, and outcome of CAD. Traditional inflammatory biomarkers such as tumor necrosis factor-alpha (TNF-*α*) [23] (Mizia-Stec et al., 2003), C-reactive protein (CRP) [24], and interleukin-6 (IL-6) [25] have been shown to predict cardiovascular events. More recently, several new nontraditional biomarkers, such as CD40 and CXCR4, have been introduced in the clinical practice.

CD40 is a 50-KD integral membrane protein of tumor necrosis factor receptor family. CD40L had been found positively expressed in the atheromatous plaque. Increasing evidence shows that the CD40-CD40L system plays a critical role in atherosclerotic disease progression and plaque destabilization [13]. Studies showed that polymorphisms in* CD40* may influence individuals' susceptibility to the development of cardiovascular events [18, 26]. Studies had reported the relationship between rs1883832 at* CD40* locus and acute coronary syndrome [27, 28]. In this study, we selected two different tag SNPs (rs1535045 and rs4239702) at the* CD40* locus to analyze the relationship between the two different tag SNPs and CAD in Chinese Han population. We found that the frequency of rs1535045-T allele in cases was higher than that in controls (*P* < 0.05). It indicates that T allele of rs1535045 at the* CD40* locus may be a susceptive factor for CAD in Chinese Han population. There are no statistical significant differences of genotypic frequencies of rs1535045 between cases and controls, but they still seemed to have the tendency (*P* = 0.056). Because our sample size is not large enough, this result may be a false-negative result. In the further study, we should increase the sample size to verify it. There was no significant difference on genotypic and allelic frequencies of rs4239702 between cases and controls before or after adjustment for traditional risk factors, which suggests that the genetic variant of rs4239702 may be not associated with CAD. Results of haplotype analysis showed that the frequency of rs4239702(C)-rs1535045(T) haplotype was associated with CAD, and it may be a risk factor of CAD in Chinese Han population (OR = 1.246, 95% CI 1.014–1.531, *P* < 0.05).

CXCR4, which is highly expressed in circulating endothelial progenitor cells (EPC), together with its ligand chemokine stromal-derived factor- (SDF-) 1 is essential to normal cardiovascular development [29]. EPC from patients with CAD or diabetes have been shown to be functionally impaired [30, 31], and the CXCR4 importantly modulates the migratory and angiogenic capacities of human EPC [29]. Based on that, we can assume that CXCR4 may be associated with CAD. In this study, through analyzing the relationship between the tag SNP (rs2228014) at* CXCR4* locus and Chinese CAD patients, we had the hypothesis inspected. Unfortunately, we did not find any significant difference on genotypic and allelic frequencies of rs2228014 between cases and controls, which suggested that the genetic variant of rs2228014 at* CXCR4* locus may be not associated with CAD in Chinese Han population.

Plasma lipid levels have been proved to be a considerable risk factor of CAD. Previous study had indicated that plasma inflammatory factors level was related to plasma lipid levels [32]. But inflammatory genes' potential impact on serum lipids in CAD patients has not been adequately studied. In this study, in order to assess whether the 3 polymorphisms had any effect on lipid, we used plasma lipid levels as quantitative traits to test the association for the three SNPs. The results showed that CAD patients with rs4239702-TT genotype had higher total cholesterol level than patients with rs4239702-CC genotype, while, in control group, there was no difference between genotypes and blood total cholesterol level; people with rs4239702-TT genotype had lower blood triglyceride level than people with rs4239702-CT genotype or rs4239702-CC genotype in control group, while, in CAD patients, there was no difference between genotypes and blood triglyceride level. It was observed that only patients with rs4239702-TT genotype at CD40 gene locus had higher blood lipid levels. It is tempting to speculate that, only in CAD patients, there were some factors interacting with rs4239702-TT genotype. Because of the interaction, patients with rs4239702-TT genotype had higher blood lipid level than other patients. But it is not clear what factors interact with rs4239702-TT genotype and how they interact with it. However, our study could not obtain the data of medications for lipid-lowering therapy, which might affect the serum lipids of subjects, to speak volumes for the impact of CD40 gene on serum lipids. In the further study, we should collect data of the detailed lipid profile and lipid-lowering agents to prove the effect of CD40 gene on serum lipids and select this as a new research aspect to explore the mechanism why genetic variants on CD40 gene can affect the occurrence of CAD.

Blood pressure and blood glucose levels associated with inflammation had been proved by many researches [33, 34]. In CAD patients, many of them were taking medicine to control blood pressure or blood glucose levels, so, in this study, we analyzed genotype association of the 3 SNPs with history of diabetes mellitus and hypertension, instead of blood pressure and blood glucose levels. As results showed, neither of them was associated with the three SNPs.

In conclusion, we found that rs1535045-T allele at the* CD40* locus may be a susceptive factor for CAD in Chinese Han population. Variant of rs2228014 at* CXCR4* locus is not associated with CAD. rs4239702(C)-rs1535045(T) haplotype was associated with CAD. Only in CAD patients, the blood lipid level of patients with rs4239702-TT genotype was higher than other patients.

## Figures and Tables

**Table 1:** Primer sequences used to genotype 3 tag SNPs with the SEQUENOM platform.

Gene	SNPs	Forward primers	Reverse primers	Extension primers
*CD40*	rs4239702	ACGTTGGATGTAATGCCTCTCAAAGGCTTG	ACGTTGGATGTGTTTGCTTGTTGAAGGCCC	CCCGCCCAGGCCTGCTCTTGA
	rs1535045	ACGTTGGATGAATGGCTCTTAGGGAACAGG	ACGTTGGATGTTCTCCACTCCTACCACAAG	CCTCTTTCCAGCTCCA

*CXCR4*	rs2228014	ACGTTGGATGCCTTTTCAGCCAACAGCTTC	ACGTTGGATGTCATCAGTCTGGACCGCTAC	GGACCGCTACCTGGCCATC

**Table 2:** Clinical characteristics of study samples.

Variable	Case (*n* = 583)	Control (*n* = 540)	*P*
Age (year)	63.75 ± 11.56	61.70 ± 12.75	0.080
Gender (female) (%)	46.2	49.9	0.218
Smoking (%)	37.7	23.5	<0.001
Drinking (%)	21.2	22.7	0.625
BMI (Kg/m^2^)	24. 12 ± 2.73	23.94 ± 3.35	0.440
WHR	0.93 ± 0.08	0.87 ± 0.08	<0.001
Hypertension (%)	56.3	29.9	<0.001
Diabetes mellitus (%)	24.0	11.8	<0.001
TC (mmol/L)	4.98 ± 1.60	4.63 ± 1.22	0.104
TG (mmol/L)	1.73 ± 1.17	1.74 ± 1.22	0.937

Age, WHR were compared by the Mann-Whitney *U* test.

BMI, TC, and TG were performed with Student's *t*-test.

Gender, smoking, drinking, hypertension, and diabetes mellitus were compared by using Pearson's chi-square test.

**Table 3:** Distribution of allelic frequencies of SNPs in case and control groups.

SNPs	Control		Case		*χ* ^2^	*P*	OR (95% CI)	*P* ^*∗*^	OR (95% CI)^*∗*^
	C	T	C	T					
rs4239702	702	376	771	395	0.250	0.617	0.96 (0.80, 1.14)	0.491	1.04 (0.93, 1.16)
rs1535045	751	319	758	390	4.405	0.036	1.21 (1.01, 1.45)	0.042	1.27 (1.01, 1.59)
rs2228014	924	156	1009	157	0.449	0.503	0.92 (0.73, 1.17)	0.660	0.97 (0.83, 1.13)

*∗*: after adjusting the risk factors (smoking, hypertension, diabetes mellitus, and WHR), compared with C allele.

**Table 4:** Distribution of genotypic frequencies of SNPs in case and control groups.

SNPs	Genotype	Control	Case	*χ* ^2^	*P*	OR (95% CI)^a^
rs4239702	T/T	58	60	0.284	0.868	1 (reference)
	C/T	260	275			1.140 (0.573–2.271)
	C/C	221	248			0.860 (0.582–1.271)

rs1535045	T/T	48	58	5.769	0.056	1 (reference)
	C/T	223	274			1.298 (0.659–2.566)
	C/C	264	242			1.030 (0.696–1.523)

rs2228014	T/T	17	10	2.504	0.286	1 (reference)
	C/T	122	137			0.597 (0.141–2.533)
	C/C	401	436			1.328 (0.855–2.063)

a: after adjusting the risk factors (smoking, hypertension, diabetes mellitus, and WHR), compared with TT genotype.

**Table 5:** Haplotype frequencies of the CD40 gene in case and control group.

Haplotype	Control	Case	*χ* ^2^	*P*	OR (95% CI)
rs4239702(C)-rs1535045(C)	369	377	2.282	0.131	reference
rs4239702(C)-rs1535045(T)	389	319	4.343	0.037	1.246 (1.014–1.531)
rs4239702(T)-rs1535045(C)	388	374	0.295	0.587	1.06 (0.866–1.297)

Test for overall association: *χ* ^2^ = 4.668, df = 2, *P* = 0.097.

**Table 6:** Genotype association of the 3 SNPs with blood total cholesterol (mmol/L) in case and control group.

Group	SNPs	TT		TC		CC		*F*	*P*
		*n*	Mean ± SD	*n*	Mean ± SD	*n*	Mean ± SD		
Case	rs4239702	60	4.95 ± 0.97^*∗*^	275	4.61 ± 1.26	248	4.58 ± 1.21	2.208	0.111
	rs1535045	58	4.45 ± 1.04	274	4.69 ± 1.25	242	4.61 ± 1.22	1.033	0.357
	rs2228014	10	4.68 ± 1.00	137	4.68 ± 1.09	436	4.61 ± 1.26	0.162	0.851

Control	rs4239702	58	5.02 ± 1.10	260	4.93 ± 1.05	221	5.11 ± 1.02	1.845	0.159
	rs1535045	48	5.22 ± 0.95	223	5.08 ± 1.08	264	4.92 ± 1.04	2.397	0.092
	rs2228014	17	4.86 ± 0.79	122	4.99 ± 1.28	401	5.03 ± 1.00	0.269	0.764

*∗*: *P* < 0.05, compared with CC genotype.

**Table 7:** Genotype association of the 3 SNPs with blood triglyceride (mmol/L) in case and control group.

Group	SNPs	TT		TC		CC		*F*	*P*
		*n*	Mean ± SD	*n*	Mean ± SD	*n*	Mean ± SD		
Case	rs4239702	60	1.78 ± 0.93	275	1.76 ± 1.05	248	1.69 ± 1.33	0.312	0.732
	rs1535045	58	1.65 ± 1.21	274	1.72 ± 1.16	242	1.74 ± 1.16	0.126	0.882
	rs2228014	10	1.64 ± 0.88	137	1.75 ± 1.06	436	1.72 ± 1.21	0.053	0.948

Control	rs4239702	58	1.27 ± 0.65^*∗*△^	260	1.81 ± 1.28	221	1.76 ± 1.24	4.744	0.009
	rs1535045	48	1.51 ± 0.77	223	1.78 ± 1.19	264	1.74 ± 1.31	0.878	0.416
	rs2228014	17	1.70 ± 0.80	122	1.72 ± 1.18	401	1.74 ± 1.25	0.027	0.973

*∗*: *P* < 0.05, compared with CC genotype.

△: *P* < 0.05, compared with TC genotype.

**Table 8:** Genotype association of the 3 SNPs with diabetes mellitus in case and control group.

Group	SNPs	TT		TC		CC		*χ* ^*2*^	*P*
		Yes (%)	No (%)	Yes (%)	No (%)	Yes (%)	No (%)		
Case	rs4239702	9 (15.0)	51 (85.0)	73 (26.6)	202 (73.4)	58 (23.4)	190 (76.6)	3.691	0.158
	rs1535045	18 (31.0)	40 (69.0)	62 (22.6)	212 (77.4)	55 (22.7)	187 (77.3)	2.101	0.350
	rs2228014	1 (10.0)	9 (90.0)	41 (29.9)	96 (70.1)	97 (22.2)	339 (77.8)	4.459	0.108

Control	rs4239702	7 (12.1)	51 (87.9)	35 (13.5)	225 (86.5)	22 (10.0)	199 (90.0)	1.406	0.459
	rs1535045	2 (4.2)	46 (95.8)	28 (12.6)	195 (87.4)	34 (12.9)	230 (87.1)	3.055	0.217
	rs2228014	0 (0)	17 (100)	14 (11.5)	108 (88.5)	50 (12.5)	351 (87.5)	2.448	0.294

**Table 9:** Genotype association of the 3 SNPs with hypertension in case and control group.

Group	SNPs	TT		TC		CC		*χ* ^2^	*P*
		Yes (%)	No (%)	Yes (%)	No (%)	Yes (%)	No (%)		
Case	rs4239702	36 (60.0)	24 (40.0)	160 (58.2)	115 (41.8)	134 (54.0)	114 (46.0)	1.228	0.541
	rs1535045	29 (50.0)	29 (50.0)	160 (58.4)	114 (41.6)	135 (55.8)	107 (44.2)	1.446	0.485
	rs2228014	4 (40.0)	6 (60.0)	78 (56.9)	59 (43.1)	245 (56.2)	191 (43.8)	1.093	0.579

Control	rs4239702	12 (20.7)	46 (79.3)	83 (31.9)	177 (68.1)	66 (29.9)	155 (70.1)	2.857	0.240
	rs1535045	14 (29.2)	34 (70.8)	68 (30.5)	155 (69.5)	79 (29.9)	185 (70.1)	0.040	0.980
	rs2228014	3 (17.6)	14 (82.4)	30 (24.6)	92 (75.4)	129 (32.2)	272 (67.8)	3.834	0.147
